# Incidence and Predictors of Imported Cases of COVID-19 in Burkina Faso

**DOI:** 10.3389/fpubh.2022.743248

**Published:** 2022-02-17

**Authors:** Mikaila Kaboré, Kongnimissom Apoline Sondo, Désiré Lucien Dahourou, Yacouba Cissoko, Issa Konaté, Abdoulaye Zaré, Brice Bicaba, Boukary Ouedraogo, Hermann Barro, Eric Arnaud Diendéré, Isabella Asamoah, Sandrine Nadège Damoue, Baperman Abdel Aziz Siri, Ismael Diallo, Peter Puplampu, Armel G. Poda, Yacouba Toloba, Sounkalo Dao, Martial Ouédraogo, Seni Kouanda

**Affiliations:** ^1^Infectious Diseases Department, Yalgado Ouedraogo Teaching Hospital, Ouagadougou, Burkina Faso; ^2^Training and Research Unit in Health Sciences, Joseph Ki-Zerbo University, Ouagadougou, Burkina Faso; ^3^Department of Biomedical and Public Health, Research Institute of Health Sciences, Bobo Dioulasso, Burkina Faso; ^4^Faculty of Medicine and Odontostomatology, University of Sciences, Techniques and Technologies of Bamako (USTTB), Bamako, Mali; ^5^Ministry of Health, Ouagadougou, Burkina Faso; ^6^Infectious Diseases Unit, Department of Medicine, Korle-Bu Teaching Hospital, Accra, Ghana; ^7^Department of Infectious Diseases, Souro Sanon Teaching Hospital, Bobo-Dioulasso, Burkina Faso

**Keywords:** COVID-19, imported cases, incidence, predictors, Burkina Faso

## Abstract

**Background:**

To limit the spread of COVID-19 due to imported cases, Burkina Faso has set up quarantine measures for arriving passengers. We aimed to determine the incidence and predictors of imported cases of COVID-19 in Burkina Faso.

**Methods:**

A prospective cohort study was performed using data from passengers arriving at the airport from April 9 to August 31, 2020. The data was extracted from the *District Health Information Software 2 (DHIS*2) platform. Cox regression was used to identify predictors of imported cases of COVID-19.

**Results:**

Among 6,332 travelers who arrived in the study period, 173 imported cases (2.7%) were recorded. The incidence rate was 1.9 cases per 1,000 traveler-days (95%CI: 1.6–2.2 per 1,000). Passengers arriving in April (Adjusted hazard ratio [aHR] = 3.56; 95%CI: 1.62–7.81) and May (aHR = 1.92; 95% CI: 1.18–3.12) were more at risk of being tested positive compared to those arriving in August, as well as, passengers presenting with one symptom (aHR = 3.71; 95% CI: 1.63–8.43) and at least two symptoms (aHR = 10.82; 95% CI: 5.24–22,30) compared to asymptomatic travelers.

**Conclusions:**

The incidence of imported cases was relatively low in Burkina Faso between April and August 2020. The period of travel and the presence of symptoms at arrival predicted the risk of being tested positive to severe acute respiratory syndrome coronavirus 2 (SARS-CoV-2). This is essential in the context of the high circulation of virus variants worldwide and the low local capacity to perform genotyping tests to strengthen the surveillance and screening capacities at the points of entry into the country.

## Introduction

In early December 2019, the first cases of infection with COVID-19 were recorded in China (1, 2). Gradually, other continents have been affected by tourism and trade-related movements of people (3–5). On March 11, 2020, WHO declared a pandemic in view of the spread of the virus around the world (6). As of October 30, 2020, there were 46,166,518 confirmed cases with 1,196,362 deaths worldwide with a case fatality of 2.6%. Africa remains one of the regions least affected by the coronavirus pandemic (7–9).

At the onset, the main driver of the pandemic's expansion was international tourists and commercial travels from China, which was the epicenter of the epidemic. Many cases of coronavirus infections have thus been associated with a travel history that suggests the notion of imported cases of COVID-19 (5, 10, 11).

Like other countries in the world, Burkina Faso has been facing this health crisis since March 9, 2020, when the first two imported cases were recorded in the country. As of March 19, 26 confirmed cases of COVID-19 were recorded (12). Since then, measures have been taken by the authorities to reduce the spread of the disease, including the closure of land, rail, and airport which took place on March 20, 2020 (13). This measure negatively impacted the country's economy, especially the livelihoods of populations. In the field of health, the COVID-19 pandemic has displayed a dysfunction of health systems with disruption of the epidemiological surveillance system (14).

The goal of this measure was to interrupt the transmission of the virus through cases imported by travelers from epidemic countries as did most countries in the world (13). From April 9, the government decided to repatriate Burkinabè as well as humanitarian aid workers and other Non-Governmental Organizations (NGOs) workers retained in countries, most of which were already in epidemics. To prevent the risk of imported cases of COVID-19, the authorities have put in place quarantine measures including 15 days of isolation upon arrival. It only concerned passengers entering by air.

Several previous studies conducted mostly in Asia reported epidemiological and clinical aspects of imported cases of COVID-19 (11, 15–18). However, little is known about the imported cases of COVID-19 in Africa. COVID-19 screening tests are carried out before and systematically on the day of departure of travelers, however, we find cases of COVID-19 upon their arrival in the countries of destination (19). Hence the interest of our study was to estimate the incidence and predictors of imported cases of COVID-19 among inbound travelers to Burkina Faso.

## Methods

### Study Site, Type, and Period

The study was conducted nationwide in Burkina Faso. There are two international airports located in the two largest cities namely Ouagadougou and Bobo Dioulasso. These cities have the highest concentration of the national urban population (80.5%) (20).

**Figure 1:**
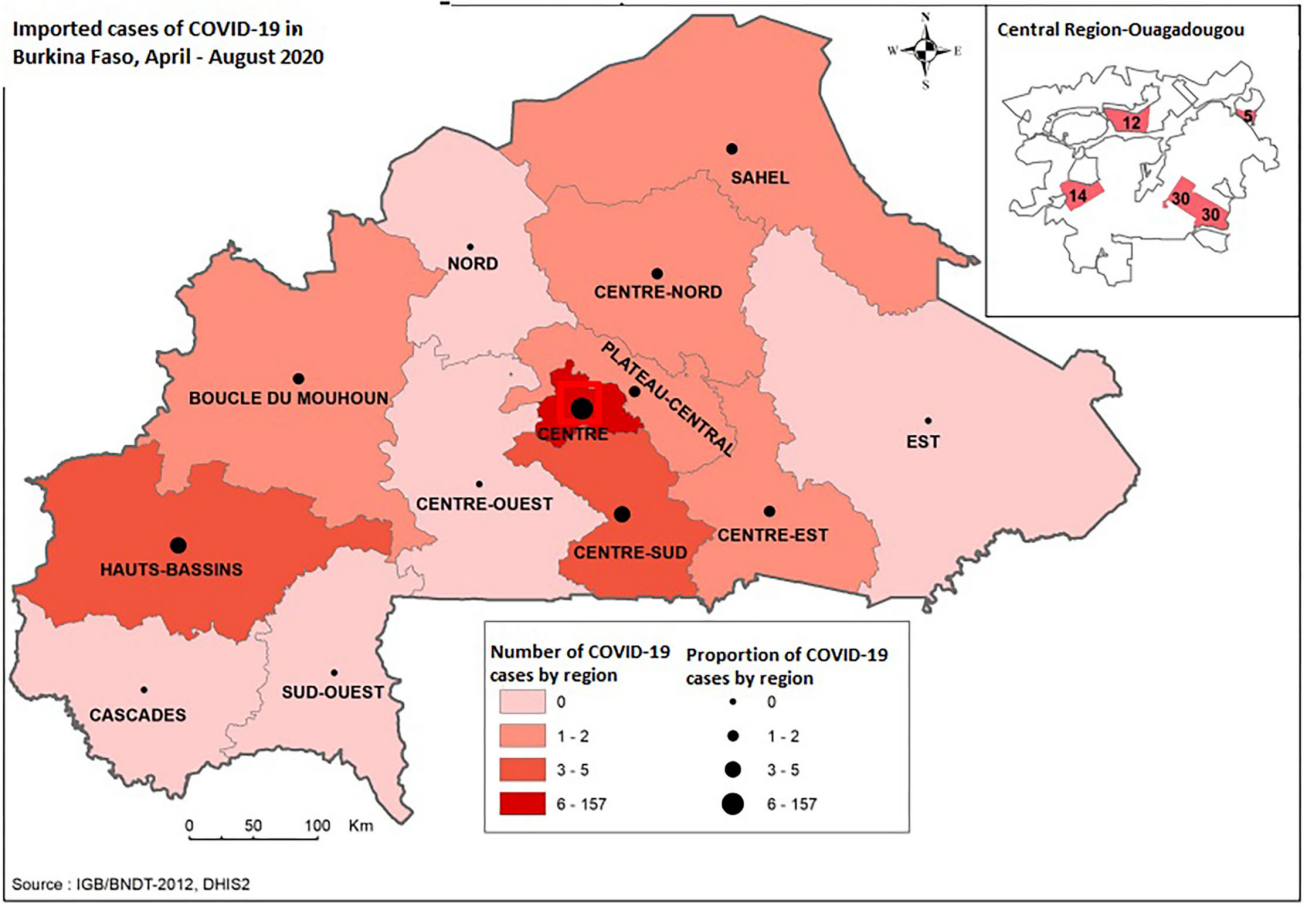
Mapping of regions of residence of imported COVID-19 cases, April-August 2020, Burkina Faso.

Figure 1 shows the 13 regions of the country. Of these, eight are expected to receive severe acute respiratory syndrome coronavirus 2 (SARS-CoV-2) positive travelers. The Center region (157 cases), Haut-Bassins (5 cases), and Centre-Sud (5 cases) were the most affected.

On arrival, passengers were systematically placed in quarantine in sites chosen for this purpose. These consisted mainly of hotels meeting the standards of amenities defined by the health authorities.

Quarantine or containment measures are restrictions of activities of suspicious persons who are not sick or the retaining of suspected baggage, containers, or goods. The objective of the quarantine is monitoring their symptoms and ensuring the early detection of cases so as to prevent the possible spread of infection (21). We conducted a prospective cohort study including arrival passengers from April 9 to August 31, 2020, with 15 days of the follow-up period.

### Study Population

All arriving passengers by international flight at both airports who were admitted to quarantine sites for two weeks during the study period were eligible in this study.

Passengers who have correctly completed the notification forms and those who were compliant with the two weeks confinement were included in this study.

### Data Source

As part of the fight against COVID-19, the Ministry of Health has set up a database to manage all data collected in relation to the disease. This database was designed using *District Health Information Software (DHIS)* version 2. The data collected in this database is follow-up information on patients, contacts, travelers, and laboratory data. We extracted data concerning incoming travelers by the airport.

### Study Variables

The study variables were socio-demographic variables (age, gender, region, country of origin), clinical variables (symptoms declared by the traveler: fever or history of fever, asthenia, cough, dyspnea, rhinitis, chest pain, headache), and biological variables (dates and analysis report of SARS-CoV-2 RT-PCR form oral and/or nasopharyngeal specimen taken on day 1, day 8, and day 15).

### Statistical Analyses

The study outcome was COVID-19 infection is defined as SARS-CoV-2 RT-PCR positive during follow-up. Study participant characteristics are presented as frequencies (percentage) for categorical variables, whereas continuous variables are presented using median and interquartile ranges (IQR). The participant's time at risk started at the arrival date and ended either at the first COVID-19 positive test or was censored at 15 days defining the end of the containment period for those with SARS-CoV-2 negative test at 1, 8, and 15 days. We estimated the cumulative incidence of COVID-19 infection with its confidence interval. Cox regression was used to estimate hazard ratios (HRs) of an RT-PCR-positive test. Variables associated with the univariate analysis with *p* < 0.25 were included in a multivariate analysis. Then, we conducted a stepwise descendant analysis. Variables were retained in the final model if significantly associated (*p* < 0.05) with the RT-PCR-positive test. All analyses were performed using SAS 9.4 (Cary, NC, USA).

## Results

### Basic Characteristics of Participants

From April 9 to August 31, 2020, 6,332 passengers arriving in Burkina Faso at the country's two international airports and compliant with the 15 days quarantine measures were included in this study. Table 1 provides the baseline characteristics of the study participants. Most of the participants were aged between 30 and 59 years. The majority was male (70%) and arrived in July and August 2020 (81.6%), from African countries (65.1%). Less than 1%, reported exposure history to symptomatic COVID-19 patients.

**Table 1:** Baseline characteristics of participants.

	**Frequency**	**Percentage**
**Gender**		
Male	4,448	70.2
Female	1,884	29.8
**Age (years) (*n* = 5,835)**		
≤ 30	1,799	30.8
30–59	3,740	64.1
≥60	296	5.1
**Month of arrival (in 2020)**		
April	70	1.1
May	447	7.1
June	633	10.0
July	1,914	30.2
August	3,268	51.6
**Continent of departure (*n* = 6,122)**		
Africa	3,876	65.1
Europe	1,266	21.2
America	368	6.2
Asia	445	7.4
**Exposure's history to symptomatic COVID-19 patient**		
No	6,307	99.6
Yes	25	0.4
**Stay in epidemic area in the last 2 weeks**		
No	526	8.3
Yes	5,806	91.7
**Number of symptoms**		
No	6,249	98.7
One	58	0.9
At least two	25	0.4

Overall, 98.7% of participants were asymptomatic at arrival. The remaining presented either one symptom (0.9%) or at least two symptoms (0.4%) during their containment.

### Incidence of Imported Cases of COVID-19 in Burkina Faso

**Figure 2:**
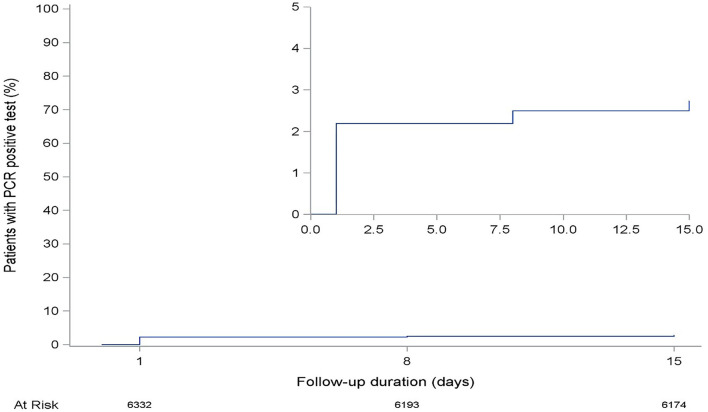
The cumulative incidence rate of severe acute respiratory syndrome coronavirus 2 (SARS-CoV-2) test positivity among airport arrival passengers, April–August 2020, Burkina Faso.

During follow-up, 173 passengers (2.7%) had at least one SARS-CoV-2 RT-PCR positive result. This represented 17.9% of 965 confirmed cases of COVID-19 during the study period. The total participant time at risk was 86,476.5 passengers-days. The incidence rate of SARS-CoV-2 infection was 1.9 cases per 1,000 passengers-day (95% CI: 1.6–2.2 per 1,000) (Figure 2). Among passengers who tested positive for SARS-CoV-2, 80% were tested positive on the first day of arrival, 11% on day 8, and 9% on day 15.

### Predictors of Imported Cases of COVID-19 in Burkina Faso

In the unadjusted analysis, gender, the period of arrival, the history of exposure to symptomatic patients two weeks before the travel, and the clinical condition of the passengers on arrival was associated with the SARS-CoV-2 RT-PCR test positivity in inbound passengers with *p* < 0.25 (Table 2).

**Table 2:** Univariate and multivariate analysis of the risk of being PCR-positive.

	***N***	**PCR positive *n* (%)**	**Unadjusted hazard ratio (95%CI)**	***p*-value**	**Adjusted hazard ratio (95%CI)**	***p*-value**
Gender				0.156		
Male	4,448	113 (2.5)	1			
Female	1,884	60 (3.2)	0.80 (0.58–1.10)			
Age (years)				0.804		
<30	1,799	49 (2.7)	0.81 (0.41–1.59)	0.533		
30–59	3,740	102 (2.7)	0.81 (0.42–1.54)	0.516		
≥60	296	10 (3.4)	1			
Month of arrival (in 2020)				<0.001		0.001
April	70	7 (10.0)	4.36 (2.01–9.46)	<0.001	3.56 (1.62–7.81)	0.001
May	447	21 (4.7)	2.03 (1.25–3.29)	0.004	1.92 (1.18–3.12)	0.008
June	633	25 (3.9)	1.70 (1.08–2.68)	0.021	1.51 (0.95–2.39)	0.076
July	1,914	44 (2.3)	0.99 (0.68–1.43)	0.951	0.96 (0.67–1.40)	0.853
August	3,268	76 (2.3)	1		1	
Continent of departure				0.977		
Africa	3,986	110 (2.8)	1			
Europe	1,301	35 (2.7)	0.98 (0.67–1.43)	0.895		
America	378	9 (2.4)	0.86 (0.44–1.71)	0.674		
Asia	458	13 (2.8)	1.03 (0.58–1.83)	0.921		
Exposure's history to symptomatic COVID-19 patient	<0.001					
No	6,307	169 (2.7)	1			
Yes	25	4 (16.0)	6.14 (2.28–16.55)			
Stay in epidemic area in the last 2 weeks	0.514					
No	526	12 (2.3)	1			
Yes	5,806	161 (2.8)	1.22 (0.68–2.19)	0.514		
Number of symptoms				<0.001		<0.001
No	6,249	159 (2.5)	1		1	
One	58	6 (10.3)	4.13 (1.83–9.32)	<0.001	3.71 (1.63–8.43)	0.002
A least two	25	8 (32.0)	13.39 (6.58–27.25)	<0.001	10.82 (5.24–22.30)	<0.001

In the multivariate analysis, the month of arrival (*p* < 0.001) and clinical conditions on arrival (*p* < 0.001) were significantly associated with SARS-CoV-2 infection. Thus, the risk of SARS-CoV-2 positivity was significantly greater among passengers who arrived in April (aHR = 3.56; 95% CI: 1.62–7.81) and May (aHR = 1.92; 95% CI: 1.18–3.12) compared to those who arrived in August.

Regarding the clinical conditions of the passengers, those who had presented one symptom and two symptoms or more during their quarantine were, respectively, 4 times (aHR = 3.71; 95% CI: 1.63–8.43) and 11 times (aHR = 10.82; 95% CI: 5.24–22.30) more likely to be positive to the SARS-CoV-2 RT-PCR compared to asymptomatic (Table 2).

## Discussion

In this cohort study conducted from April 9 to August 31, 2020, the incidence of imported cases of COVID-19 was 1.9 cases per 1,000 passengers-day. Moreover, the strong predictors of imported cases were the period of travel and passengers' clinical condition on arrival and during the quarantine measure.

In this study, COVID-19 incidence was relatively low. This could be explained by the origin of our participants. Indeed, the majority of passengers (65.1%) arrived from African countries where the epidemic burden was less important than other continents at this period (8). Moreover, the lower incidence may be due to reduced international air travel (22). That was the case in our study where the air borders were closed until the end of July with a restriction of flights to Burkina Faso. In addition, we can assume that the implementation of systematic screening before the departure, plus screening on the day of travel in some airports could explain the low incidence of SARS-CoV-2 positive in travelers on arrival during our study period (19). However, despite this screening device on departure, we have notified positive cases on arrival, which leaves us perplexed and implies the need to systematically have a SARS-CoV-2 test on arrival for all passengers.

The proportion of imported cases was 17.9% of all the confirmed cases of COVID-19 from April 9 to August 31, 2020. In contrast to our study, a study analyzing the epidemiological and clinical aspects of imported cases of COVID-19 nationwide in Taiwan reported that 86.1% out of all 373 confirmed cases of COVID-19 from January 21 to April 6, 2020 were imported (15). This study took place <3 months after the onset of the pandemic, compared to our study which lasted 5 months. The probable lack of systematic screening for COVID-19 before departure could explain the high proportion of cases in their study. Moreover, the density of the air traffic for tourist or commercial reasons between Taiwan and other high epidemic burden countries like China at a beginning of the epidemic could explain this high frequency of imported cases in this study (23). Furthermore, our study began in April, therefore not taking into account the first imported cases recorded in the country before the border closure (24). In Spain, which was one of the European countries most affected by the COVID-19 epidemic, a mild proportion of imported cases were reported. It was estimated at around 0.08% based on the total number of confirmed cases of COVID-19 from May to December 2020. Considering this figure, the authors claim that the share of imported cases in the dynamics of disease's spread was relatively low so could not justify the strict and untargeted restrictions in countries with a high incidence of COVID-19 (25).

We identified two predictors of imported cases of COVID-19: the period of arrival and passengers' clinical condition on arrival. Indeed, those who arrived in April and May were, respectively, four and two times more likely to have a positive SARS-CoV-2 RT-PCR test, compared to those who arrived in August. Both these months were the period of epidemic peaks in many countries, including Burkina Faso (26) with more transmissions and a high number of confirmed cases. Despite the high flow of travelers from August due to the reopening of air borders, the risk of imported cases was low compared to other months. The measure of entrance restriction only cannot prevent the importation of COVID-19 cases in a country. Vigorous quarantine measures and screening of cases are essential to avoid the spread of the infection in the community.

Passengers who presented one symptom and those with two or more symptoms were, respectively, 4 times and 11 times more likely to test positive for COVID-19 compared to asymptomatic. In other words, the more symptomatic the passengers were on arrival, the greater the risk of COVID-19 test positivity. These results confirm the fact that the presence of symptoms known compatible with COVID-19 in any individual including passengers must require adequate measures to be taken to reduce the risk of transmission of the disease through systematic quarantine and surveillance, while doing the necessary to confirm the diagnosis. This highlights the issue of self-quarantine at home with the inability to monitor movements of the suspected cases, and probable community spread. Moreover, there is the problem of screening symptomatic passengers on departure and their flight ban to avoid future contamination. In the study carried out in Taiwan, only symptomatic travelers and passengers from epidemic countries were systematically screened with home quarantine. This strategy was related to several contaminations in the entourage of travelers (15). In our study, most of the passengers including the imported cases were asymptomatic (98.7%). If the COVID-19 test was applied only to symptomatic travelers, this measure would generate many cases of contamination in the community. Previous studies found that asymptomatic carriers are infectious and therefore can potentially transmit the disease and claim particular attention in their identification and monitoring (27, 28). The quarantine measures put in place may therefore have prevented the further spread of the epidemic in the community.

We noticed that the departure continent did not predict the COVID-19 test result among inbound travelers to Burkina Faso, although Asia and America were the continents that reported the highest prevalence of cases of COVID-19 at this period.

Our study has some limitations. First, we did not include passengers who have incorrectly completed the notification forms, those who were not compliant with the 2 weeks quarantine, and those who escaped from quarantine in this study. This selection bias might have underestimated the incidence of the COVID-19 imported cases. In fact, these passengers could be more at risk, which would explain their non-compliance with the quarantine measures. Except for fever, the other clinical signs were based on the passenger's statement. This could lead to an information bias if the passenger reports wrong information for fear of quarantine and stigmatization.

Notwithstanding these limitations, our study's strength resided firstly in the fact that it was performed at the national level and cover a period enough to appreciate the possible fluctuations due to the dynamics of the COVID-19 pandemic. Moreover, this is the first to report the incidence of imported cases in West Africa and to identify the factors associated with COVID-19 imported cases among international travelers during this pandemic period. These data are useful for the best preparedness the response to future challenges related to infectious disease outbreaks.

## Conclusion

The COVID-19 pandemic poses a major threat to public health. In Burkina Faso, there has been a relatively low incidence rate of imported cases of COVID-19. The period of travel and the presence of symptoms at arrival predicted the risk of being tested positive for SARS-CoV-2 infection during the quarantine. It is therefore imperative to strengthen the surveillance and screening capacities at the entrance gates, regardless of the clinical symptom to break the chain of transmission related to imported cases. This is essential in a context of high circulation of virus variants worldwide and low local capacity to perform genotyping tests.

## Data Availability Statement

The raw data supporting the conclusions of this article will be made available by the authors, without undue reservation.

## Ethics Statement

The protocol was approved by the Ethics Committee for Health Research in Burkina Faso (Deliberation No. 2020-9-211). The Ministry of Health has also given its approval for the use of data from the *DHIS2* database. The confidentiality, as well as the anonymity of travelers, were respected during data processing.

## Author Contributions

MK, KS, DD, SD, and SK developed the study concept. BO, HB, and BS managed the data with the software. BB and MO authorized the study performance. MK, KS, DD, and SK analyzed and interpreted the data. MK, KS, DD, and SD drafted the manuscript. YC, IK, AZ, IA, SD, ID, ED, PP, AP, YT, and SD contributed to the writing and revision of the manuscript. All authors approved the final version of the manuscript for submission.

## Conflict of Interest

The authors declare that the research was conducted in the absence of any commercial or financial relationships that could be construed as a potential conflict of interest.

## Publisher's Note

All claims expressed in this article are solely those of the authors and do not necessarily represent those of their affiliated organizations, or those of the publisher, the editors and the reviewers. Any product that may be evaluated in this article, or claim that may be made by its manufacturer, is not guaranteed or endorsed by the publisher.
